# Synergic Effect of *α*-Mangostin on the Cytotoxicity of Cisplatin in a Cervical Cancer Model

**DOI:** 10.1155/2016/7981397

**Published:** 2016-12-08

**Authors:** Jazmin M. Pérez-Rojas, Raquel González-Macías, Jaime González-Cortes, Rafael Jurado, José Pedraza-Chaverri, Patricia García-López

**Affiliations:** ^1^División de Investigación Básica, Instituto Nacional de Cancerología, Av. San Fernando #22, Apartado Postal 22026, Tlalpan, 14000 Mexico City, Mexico; ^2^Facultad de Química, Universidad Nacional Autónoma de México, 04510 Mexico City, Mexico

## Abstract

Cervical cancer is the second leading cause of death among Mexican women. The treatment with cis-diamminedichloroplatinum (II) (CDDP) has some serious side effects.* Alpha*-mangostin (*α*-M), has a protective effect against CDDP-induced nephrotoxicity, as well as antioxidant, antitumor, and anti-inflammatory properties. Hence, we explored the in vitro and in vivo effect of *α*-M on human cervical cancer cell proliferation when combined with CDDP. In vitro, The cytotoxic effect of *α*-M and/or CDDP was measured by the 3-(3,5-dimethylthiazol-2-yl)2,5-diphenyltetrazolium assay. Meanwhile, apoptosis, reactive oxygen species (ROS) production, and the cell cycle were determined with flow cytometry. For *α*-M+CDDP treatment, both a coincubation and preincubation scheme were employed. In vivo, xenotransplantation was performed in female athymic BALB/c (nu/nu) mice, and then tumor volume and body weight were measured weekly, whereas *α*-M interfered with the antiproliferative activity of CDDP in the coincubation scheme, with preincubation with *α*-M+CDDP showing significantly greater cytotoxicity than CDDP or *α*-M alone, significantly inhibiting average tumor volume and preventing nephrotoxicity. This effect was accompanied by increased apoptosis and ROS production by HeLa cervical cancer cells, as well as an arrest in the cell cycle. These results suggest that *α*-M may be useful as a neoadjuvant agent in cervical cancer therapy.

## 1. Introduction

Despite efforts to improve early detection of cervical cancer, it is still one of the leading causes of death worldwide for women over 20 years of age. In Mexico, annually there are about 68,000 cases [1, 2]. Patients with this kind of cancer have three options for treatment: surgery, radiotherapy, chemotherapy, or a combination. In chemotherapy, cis-diamminedichloroplatinum II (CDDP) is the most commonly used drug [3]. CDDP forms adducts between nitrogen bases induces apoptosis and generates reactive oxygen species (ROS) [4, 5]. Unfortunately, CDDP has several secondary effects, mainly in the kidney and brain, and leads to chemoresistance [6, 7]. Therefore, it is necessary to continue searching for new anticancer compounds and/or adjuncts to current treatments.

Nowadays, people consume many natural products to obtain beneficial health effects from their bioactive compounds. One example is *α*-mangostin (*α*-M), a prenylated xanthone isolated from the mangosteen tree (*Garcinia mangostana* Linn.). Many in vitro and in vivo studies carried out on *α*-M have reported antioxidant and antitumorigenic effects, among other properties [8, 9]. In the last few years, studies on the anticancer properties of *α*-M and other compounds derived from the mangosteen tree have shown excellent results, mostly demonstrating their efficacy and low toxicity.

Since the sale of juices and extracts of these compounds is not restricted, they are freely consumed. Sometimes, patients under chemotherapy use such products, unaware of the effect that may result from combining them with their treatment. There are few studies on the possible impact of the natural compounds from the mangosteen tree on the antiproliferative and secondary effects produced by CDDP. Hence, we decide to conduct such a study with *α*-M because it is known to improve the antiproliferative effect of CDDP while minimizing its secondary effects [9–11]. At the same time, it minimally alters normal cells [12–14].

Previously, we demonstrated that *α*-M has a renoprotective effect on CDDP-induced nephrotoxicity. This effect was mediated by preventing an increase in ROS, tumor necrosis factor-*α*, and transforming growth factor *β*, without showing changes in the pharmacokinetics of CDDP [15]. Since *α*-M has antiproliferative and renoprotective effects, the aim of the present study was to evaluate the possibility that the combination of this compound with CDDP could improve cytotoxicity and diminish secondary effects. Thus, we determined the cytotoxic effect of CDDP+*α*-M in vitro as well as in an in vivo cervical cancer model.

## 2. Material and Methods

### 2.1. Materials

*α*-M was purified as previously described from mangosteen pericarp [16], purchased from DNP International Inc. (Whittier, CA, USA). CDDP, 3-(3,5-dimethylthiazol-2-yl)2,5-diphenyltetrazolium (MTT), and 5-(and-6)-carboxy-2′,7′-dichlorodihydrofluorescein diacetate (carboxy-H_2_DCFDA) were obtained from Sigma-Aldrich Co. (St. Louis, MO, USA). Dimethyl sulfoxide (DMSO) was obtained from MP Biomedicals (Solon, OH, USA). All reagents for flow cytometry were obtained from Millipore Inc. (Darmstadt, DE, USA). All other reagents used were obtained from commercial sources.

### 2.2. Cell Culture

The human cervix adenocarcinoma cell line (HeLa) was obtained from American Type Culture Collection (Manassas, VA, USA). Cells were routinely maintained in Dulbecco's modified Eagle's medium (DMEM) supplemented with 10% fetal bovine serum (Gibco BRL) and incubated at 37°C under an atmosphere of 5% CO_2_ at high humidity.

### 2.3. Cell Viability Assay

Mitochondrial function was estimated by the MTT assay, which is based on the reduction of a tetrazolium salt by the mitochondrial dehydrogenase enzyme in viable cells. After treatment, the medium was removed. 5 mg/mL MTT was diluted in DMEM without phenol red (1 : 9) and 100 *μ*L of reagent was added, and then the solution was incubated for 1 h. Formazan dye was dissolved with 2-propanol acid, and optical density was measured using an ELISA reader at *λ* 570 nm. The results are expressed as the percentage of MTT reduction in relation to control values.

### 2.4. Effect of *α*-M and CDDP on Cell Viability

The effect of *α*-M and CDDP on cell viability was determined using the MTT assay. After seeding 1 × 10^4^ cells/well in culture medium in 96-well plates, *α*-M (0 to 80 *μ*M) or CDDP (0 to 120 *μ*M) was added, cells were incubated for 24 h, and finally cell viability was quantified. The percentage of growth inhibition was calculated and the concentration was determined at which each drug achieved 50% growth inhibition (IC_50_). A theoretical isobologram was then constructed to determine the concentration that would be used for the drug combinations subsequently evaluated in two different schemes: coincubation and preincubation. Whereas in the former scheme both agents were coincubated for 24 h, in the latter the cells were exposed to *α*-M for 24 h and then incubated with CDDP for an additional 24 h. The results are expressed as the percentage of MTT reduction. The experiment was conducted in triplicate in independent experiments.

### 2.5. Isobologram

The isobologram method was used to determine the inhibition of cell viability in function of the interaction between the two drugs [17, 18]. The IC_50_ of CDDP alone was plotted on the abscissa and of *α*-M on the ordinate. The combination of these two compounds, having additive effects, should fall on a straight line connecting these two points. While points above the line represent antagonism, those below the line show synergism.

### 2.6. Drug Interaction Analysis

The combination index (CI), calculated for data analysis of combined drug treatment, was determined by median-effect principle derived by Chou [19, 20]. The equation correlates the drug dose and cytotoxic effect in the following way: CI = (*D*_1_/*D*_*x*1_) + (*D*_2_/*D*_*x*2_) + *α*(*D*_1_ *D*_2_/*D*_*x*1_ *∗D*_*x*2_), where *D*_1_ and *D*_2_ represent the concentrations used in the combined treatment, while *D*_*x*1_ and *D*_*x*2_ are single treatment concentrations giving the same response as *D*_1_ and *D*_2_, respectively. The factor *α* indicates the type of interaction: *α* = 0 for similar mechanisms of action and *α* = 1 for independent modes of action (*α* = 1 was herein employed). CI = 1 indicates an additive effect, CI < 1 synergism, and CI > 1 antagonism.

### 2.7. Determination of Reactive Oxygen Species (ROS) Production by Flow Cytometry

Cells (2 × 10^5^) were seeded in 6-well plates and then treated by using the coincubation or preincubation scheme. The cells were later washed with phosphate-buffered saline solution (PBS) and harvested with PBS-EDTA, followed by centrifuging at 2,000 rpm for 8 minutes and resuspension in PBS. For ROS detection, they were treated with 10 *μ*M of the fluorescent marker 5-(and-6)-carboxy-2′,7′-dichlorodihydrofluorescein diacetate (carboxy-H_2_DCFDA, Sigma-Aldrich) [21] for 30 minutes in the dark. Flow cytometry was performed over 10,000 acquired events with InCyte software (Merck Millipore, Darmstadt, DE), with hydrogen peroxide (1 mM per 24 h) used as the positive control. The experiment was carried out in triplicate.

### 2.8. Determination of Apoptosis by Flow Cytometry

Cells (2 × 10^5^) were seeded in 6-well plates and then treated by using the coincubation or preincubation scheme. The cells were later washed with PBS and harvested with PBS-EDTA, followed by centrifugation at 2,000 rpm for 8 minutes. Cells were resuspended in PBS and then treated with Guava Nexin Reagent to measure apoptosis at room temperature for 20 minutes in the dark. Flow cytometry was performed over 10,000 acquired events with Guava Nexin software (Merck Millipore, Darmstadt, DE). Early and late apoptosis was determined, and the results are expressed as the total percentage of apoptosis. Experiments were carried out in triplicate.

### 2.9. Determination of the Cell the Cycle by Flow Cytometry

Cells (2 × 10^5^) were seeded in 6-well plates and then treated by using the coincubation or preincubation scheme. Afterwards, the cells were washed with PBS and harvested with PBS-EDTA, followed by centrifugation at 2,000 rpm for 8 minutes. They were then resuspended in PBS and 70% ethanol and incubated at 4°C overnight. Thereafter, to measure the cell cycle, cells were washed with PBS and then treated with Guava Cell Cycle Reagent at room temperature for 30 minutes in the dark. Flow cytometry was performed over 10,000 acquired events with cellCycle software (Merck Millipore, Darmstadt, DE), and cell cycle phases were analyzed. Experiments were carried out in triplicate.

### 2.10. In Vivo Experiments

Female BALB/c mice (nu/nu, 20–25 g body weight) were provided by UPEAL/vivarium of Metropolitan University (Mexico City, Mexico). The procedures for animal care and use were approved by the Ethics Committee of the Instituto Nacional de Cancerología (INCan, Mexico City, Mexico). The mice had free access to sterile water and food and were kept in a pathogen-free environment (Allentown Inc., USA) at 25°C and 70% humidity. All mice were handled in accordance with the Mexican Guide (NOM-062-ZOO-1999) and the Committee for Updating the Guide for the Care and Use of Laboratory Animals [22]; Biological hazardous residues were discarded according to the corresponding guide (NOM-087-ECOL-SSA1-2001).

### 2.11. Experimental Design

All mice were subcutaneously inoculated in the back with 5 × 10^6^ HeLa (cervical cancer) cells. Once the tumor reached about 150 mm^3^, mice were divided into the following four groups: (1) control, orally administrated 0.5% carboxymethyl-cellulose, (2) the *α*-M treatment, was treated orally with 12.5 mg/Kg (suspended in 0.5% carboxymethyl-cellulose), (3) the CDDP treatment, intraperitoneally injected with CDDP (3 mg/Kg), and (4) the CDDP+*α*-M treatment, administered at the doses and by the routes aforementioned for these compounds. *α*-M was administrated for four days before injecting CDDP once a week for four weeks. *α*-M and CDDP dose used in the present study was chosen according to previous experiments performed in the laboratory [15, 23]. The tumors were measured in two dimensions with a caliper, and tumor volume was calculated by the following equation: *V* = (*π*/6)((width)^2^ *∗*length) [24]. Cell doubling time (CDT) was calculated by the following formula: CDT = (days of treatment)/((Log (final tumor) – Log (initial tumor))/Log 2) [25, 26]. Tumor and body weight were measured once a week for 10 weeks. At the end of the experiment the mice were placed in metabolic cages for 24 h, after which time animals were anaesthetized with a mix of isoflurane/oxygen 3%. Blood and urine sample were collected and frozen at −80°C to await processing.

### 2.12. Renal Toxicity

Blood urea nitrogen (BUN) levels were measured with an autoanalyzer (Beckman Coulter laboratory analyzer AU680 Chemistry System). Urine protein was analyzed by using the bicinchoninic acid assay [27] with bovine serum albumin (BSA) as the standard. Kidney injury molecule 1 (KIM-1) was measure with an immunoassay kit (Cloud-Clone Corp. TX, USA) following the manufacturer's instructions.

### 2.13. Statistics

Values are expressed as the mean ± SD of at least three independent experiments. The tumor volume was analyzed by ANOVA for repeated measures followed by the Student Newman-Keuls comparison test. The other parameters were evaluated using one-way ANOVA and the Student Newman-Keuls correction for multiple comparisons with GraphPad Prism 4 software (San Diego, CA). Differences were considered significant at *p* ≤ 0.05.

## 3. Results

### 3.1. Cytotoxicity of *α*-M and CDDP on Human Cervical Cancer Cells

The IC_50_ for CDDP was 29.7 ± 1.3 *μ*M and for *α*-M 19.1 ± 1.9 *μ*M. With these data, an isobologram was built to obtain the concentrations for the combined drug treatment that should give an additive effect, as well as those that theoretically give a synergist effect (data not shown).

**Figure 1:**
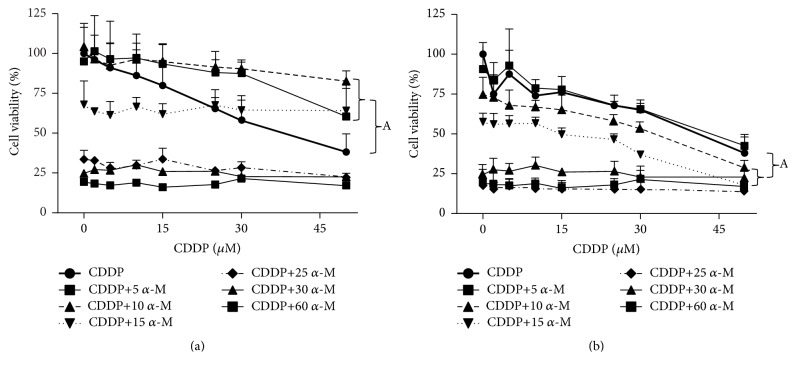
Cell viability. Using the combined treatment with *α*-M+CDDP, the cytotoxic effect on HeLa human cervical cancer cells was determined with (a) the coincubation scheme and (b) the preincubation scheme. The values are expressed as a percentage of viability (100%) found in the absence of the drug. Each point shows the mean ± SD of three independent experiments. A versus CDDP group; *p* ≤ 0.05.

The cytotoxic effect of CDDP in HeLa cells is depicted in Figure 1 for coincubation (Figure 1(a)) and preincubation (Figure 1(b)). CDDP and *α*-M showed a cytotoxic effect in a concentration-dependent manner. For the coincubation scheme, the combined treatment with *α*-M+CDDP provided greater protection of the cell against CDDP cytotoxicity at low concentrations of *α*-M (5 to 15 *μ*M) compared to the high concentration. This effect of the combined treatment at 5 to 15 *μ*M of *α*-M was statically significant in relation to the CDDP treatment alone. In the preincubation scheme, on the other hand, the combined treatment decreased cell viability in a concentration-dependent manner for both compounds. Moreover, the concentration of 10 and 15 *μ*M of *α*-M also showed statistical significance with respect to the CDDP treatment alone (Figure 1(b)).

### 3.2. Drug Interaction of *α*-M and CDDP

Once the IC_50_ was obtained for *α*-M and CDDP separately, the effect of the combined treatment on the percentage of cell viability was determined. In the coincubation scheme, neither of the combinations produced a synergistic effect. With the preincubation scheme, contrarily *α*-M increased CDDP cytotoxicity in HeLa cells (Table 1). Based on these results, we chose the concentration of 10 and 15 *μ*M of *α*-M and 2 *μ*M of CDDP for subsequent experiments. For both the preincubation and coincubation schemes, the higher concentrations of *α*-M (25 *μ*M or greater) combined with any concentration of CDDP led to a cell viability that was not different from treatment with *α*-M alone (Figure 1).

### 3.3. Effect of *α*-M and CDDP on ROS Production

**Figure 2:**
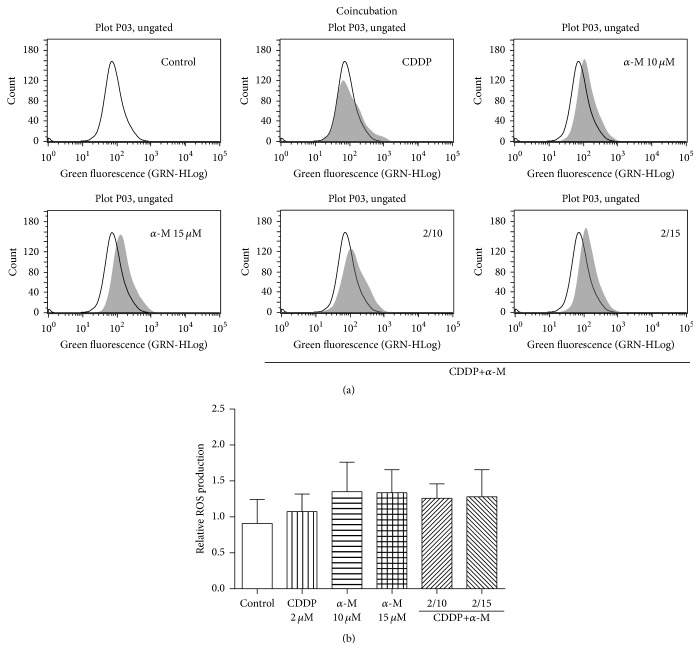
ROS generation. (a) Flow cytometry of ROS production with representative histograms. The empty histogram denotes the control group. (b) The effect of CDDP, *α*-M, and the combined treatments on ROS generation with the coincubation scheme. Each bar represents the mean ± SD of four independent experiments.

**Figure 3:**
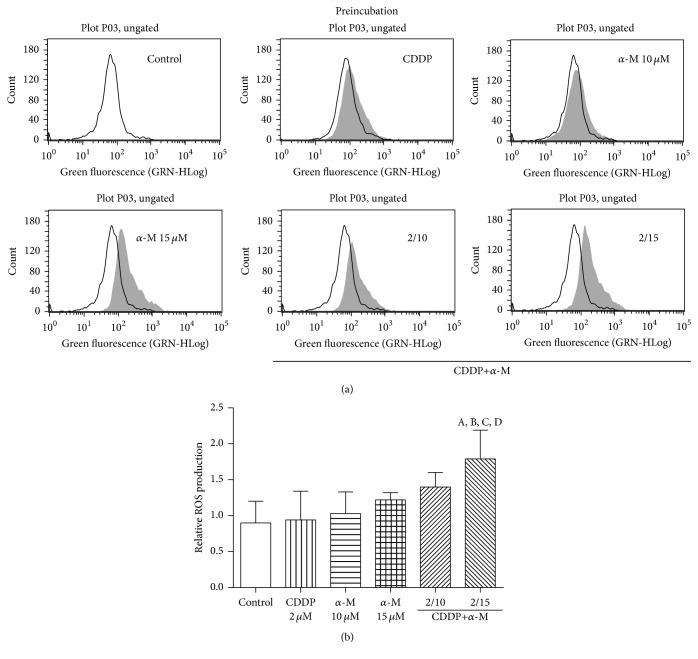
ROS generation. (a) Flow cytometry of ROS production with representative histograms. The empty histogram denotes the control group. (b) The effect of CDDP, *α*-M, and the combined treatments on ROS generation with the preincubation scheme. Each bar represents the mean ± SD of four independent experiments. A versus control group; B versus CDDP group; C versus *α*-M 15 *μ*M group; D versus CDDP+*α*-M 2/10 group; *p* ≤ 0.05.

In the coincubation scheme, no concentration of *α*-M or CDDP alone nor any combined treatment could significantly increase ROS production (Figure 2). With the preincubation scheme, the combination of 2 *μ*M of CDDP plus 15 *μ*M of *α*-M significantly elevated ROS generation compared to the control group and the individual treatments (Figure 3).

### 3.4. Effect of *α*-M and CDDP on Apoptosis

**Figure 4:**
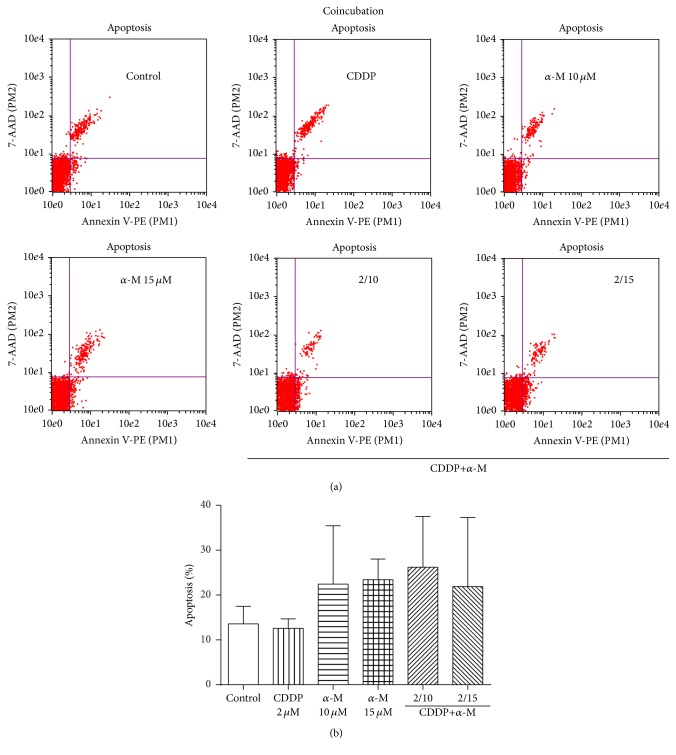
Apoptosis. (a) Dot blots representative of the coincubation scheme. (b) Flow cytometry analysis of the percentage of apoptosis found with the coincubation scheme in the control, CDDP, *α*-M, and combined treatment groups. Each bar represents the mean ± SD of four independent experiments.

**Figure 5:**
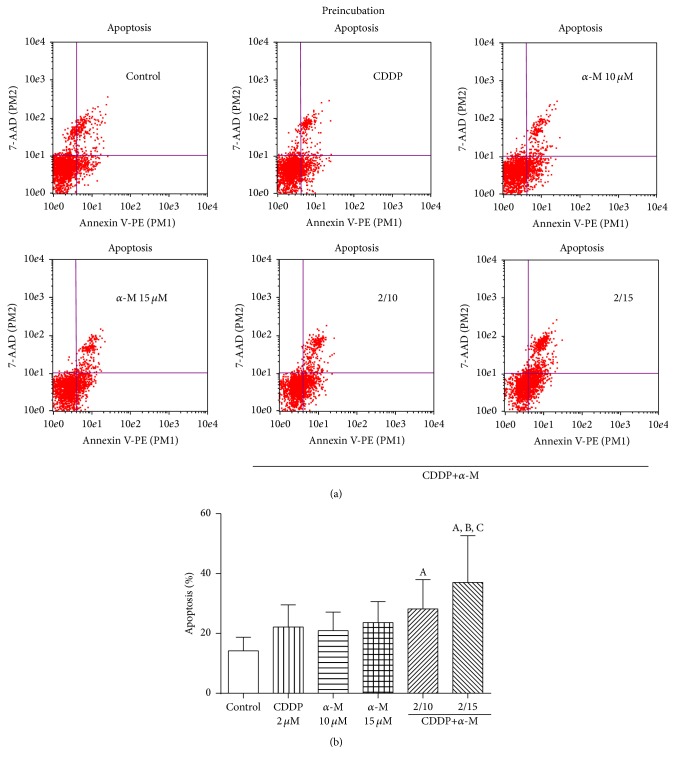
(a) Dot blots representative of the preincubation scheme. (b) Flow cytometry analysis of the percentage of apoptosis with the preincubation scheme in the control, CDDP, *α*-M, and combined treatment groups. Each bar represents the mean ± SD of four independent experiments. A versus control group; B versus CDDP group; C versus *α*-M 15 *μ*M group; *p* ≤ 0.05.

In the apoptosis analysis, we did not find a statistically significant difference between any of the groups in the coincubation scheme (Figure 4). In the preincubation scheme, however, both combined treatments significantly increased the percentage of apoptosis in relation to the control group (Figure 5). In fact, the combination of CDDP and 15 *μ*M of *α*-M is different from the treatment with *α*-M alone at this same concentration. These data correlate with the decrease in cell viability found when using this combined treatment in the preincubation scheme.

### 3.5. Effect of *α*-M and CDDP on the Cell Cycle

The distribution of the cell cycle was analyzed when cells were treated with *α*-M, CDDP and *α*-M+CDDP (considering the coincubation and preincubation schemes) (Table 2). CDDP alone increased the S and G2/M phase, although it only was significant in the preincubation scheme. Contrarily, *α*-M at both concentrations gave similar results to those found in the control group. On the other hand, the combined treatments increased the arrest in G2/M phase, like the CDDP group but in some cases did not reach statistical significance.

### 3.6. In Vivo Effect of the *α*-M+CDDP Treatment

**Figure 6:**
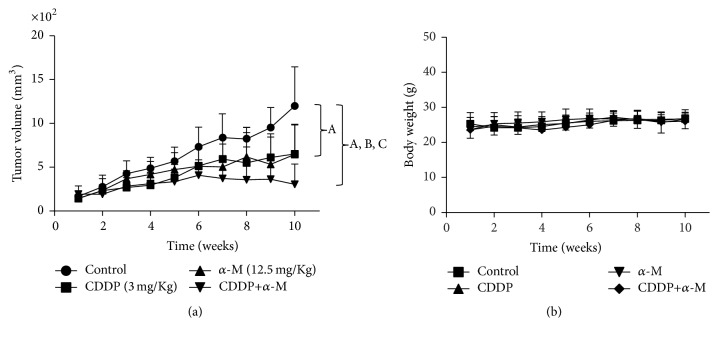
(a) Time course of tumor volume growth in mice after a subcutaneous implant of HeLa cells that were treated with the vehicle (control), CDDP, *α*-M, and CDDP+*α*-M. (b) Body weight, measured once per week, of mice treated with the vehicle, CDDP, *α*-M, and CDDP+*α*-M. Each bar represents the mean ± SD of six to eight animals. A versus control group; B versus CDDP group; C versus *α*-M group; *p* ≤ 0.05.

**Figure 7:**
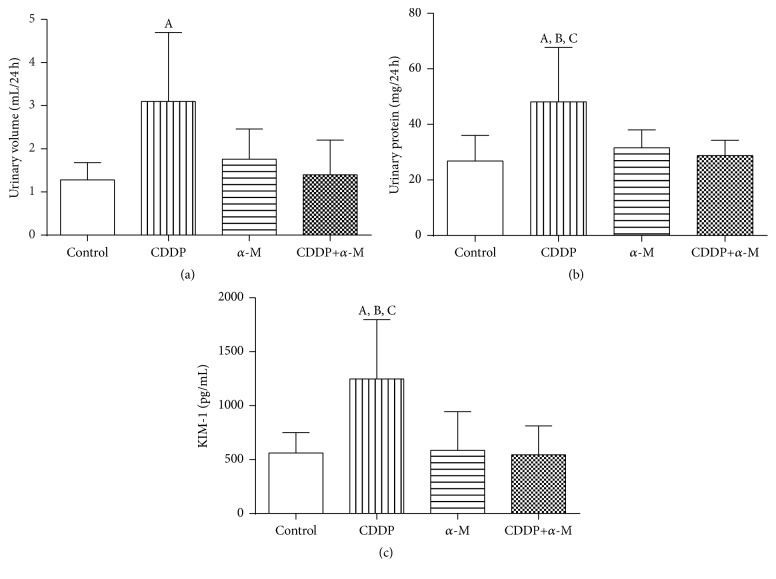
Markers of nephrotoxicity for all groups. (a) Urinary volume. (b) Urinary protein. (c) Kidney injury molecule 1 (KIM-1). CDDP: cisplatin; *α*-M: alpha mangostin. Each bar represents the mean ± SD of six to eight animals. A versus control group; B versus *α*-M group; C versus CDDP+*α*-M group; *p* ≤ 0.05.

Upon finding that *α*-M had a synergistic effect with CDDP in cell cultures and that the pretreatment scheme was more effective, we decided to conduct experiments on mice using only this scheme. The combined treatment proved to be more effective for controlling the tumor growth rate than the vehicle (control) and the individual treatments (Figure 6(a)). Additionally, for mice treated with the combined treatment versus animals in the control group, the mean cell doubling time in tumors was 9 days versus 4 days (Table 3), a difference that was statistically significant. We then measured systemic and renal toxicity in all groups, finding no significant difference in the body weight of mice between any of the studied groups (Figure 6(b)). Moreover, there were no significant changes in the BUN levels between any of the studied groups (data not show). Treatment with CDDP alone significantly increased urinary volume, urinary protein, and KIM-1 compared to the control. Conversely, *α*-M+CDDP treatment led to no increase in those parameters (Figures 7(b) and 7(c)).

## 4. Discussion

Cervical cancer is a worldwide public health problem. CDDP is the gold standard of chemotherapy for this type of cancer, although it has several side effects. Therefore, new drugs and/or modalities of treatment should be explored [28].

Over recent years in Mexico and around the world, there has been an increase in cancer research and a greater focus on new therapeutic strategies and detection methods [29]. It has been reported that a therapeutic and preventive effect can be achieved for several types of cancer with phytochemical derivatives of food or food sources, like capsaicin (peppers) [30], curcumin (curcuma) [31], resveratrol (grapes) [32], lycopene (tomatoes) [33], cinnamon essential oil [34], and others [35].

Regarding *α*-M, it is known to have antioxidant, antitumorigenic, anti-inflammatory, and antibacterial properties [8]. This compound has been under study in the las few years because of these properties as well as its antiproliferative effect. It has been demonstrated that *α*-M decreases cellular proliferation in vivo and in vitro with leukemia [36–38], colon cancer [12, 39], prostate cancer [13], and breast cancer [40]. Moreover, our group previously reported that *α*-M has a renoprotective effect against damage induced by CDDP nephrotoxicity [15]. To our knowledge, the possible adjuvant effect of *α*-M when combined with CDDP has not been previously described.

The perfect combination of drugs would be one generating synergism against cancer cells without increasing systemic toxicity. A synergistic effect refers to a combination of drugs whose effect is numerically better than that obtained by either of its components used individually [18]. The US Food and Drug Administration (FDA) has already approved the combination of CDDP with adjuvant drugs to improve the efficacy of treatment and the health of patients [41].

The mechanism of action of CDDP in the cell is the formation of platinum-DNA adducts and the inhibition of cell replication and transcription, provoking cell cycle arrest and then cell death [42]. Additionally, CDDP causes apoptotic cell death in the proximal tubular cell, which has been attributed to the generation of ROS [14]. In the present study, we demonstrated that CDDP decreased cell viability in a dose-dependent manner (Figure 1) and induced cell cycle arrest in the G2/M phase (Table 2).

The mechanism of cell death stimulated by *α*-M is not completely clear. Among the mechanisms reported is the inhibitory effect on human topoisomerases I and II, proteins that are necessary for chromosome segregation in the daughter cell. Thus, *α*-M suppresses cell proliferation, leading cells to apoptosis [43]. Another mechanism is the inhibition of CDK4 kinase, which restricts progression of the cell cycle [13]. *α*-M has also been associated with cell cycle arrest in the G2M phase by regulating expression of cdc2 cyclin and p27 [44]. Recently, Aisha and coworkers [45] found that *α*-M induces apoptosis by several mechanisms, such as through the Myc/Max and MAPK/ERK signaling pathways and the downregulation of the NFkB pathway. In this study, we designed two experimental schemes to study several mechanisms for both drugs.

We established that the IC_50_ of *α*-M in HeLa culture cells was 19.7 ± 1.0 *μ*M, similar to the value determined by Mizushina and coworkers [43]. This value is lower than the IC_50_ for CDDP (29.7 ± 1.3 *μ*M). However, when *α*-M was coincubated with CDDP, the percentage of cell viability did not decrease more than 60 percent (Figure 1(a)), indicating that low concentrations of *α*-M protect the cells against CDDP damage. In this sense, Aisha and coworkers [45] reported that at low concentrations, *α*-M significantly reduced CDDP cytotoxicity on colorectal carcinoma cells. However, when the cells were exposed first to *α*-M and then CDDP, the response was contrary. At almost all concentrations tested, the percentage of cell viability was lower for the preincubation than coincubation scheme (Figure 1(b)). After exposure to the drugs, the combination index was calculated (Table 1).

In the coincubation scheme, *α*-M interferes with the cytotoxicity activity of CDDP, which was probably the reason that changes were not observed in ROS generation. In the preincubation scheme, most combinations showed an additive effect. These results suggest that at low concentrations, *α*-M only exerts cytotoxic effects if it is administered before cancer cells are exposed to CDDP. In both schemes, any concentration of *α*-M up to EC_50_ kills cancer cells (Figure 1). This indicates the dominance of the cytotoxic effect induced by *α*-M over CDDP.

Upon finding that *α*-M enhanced the cytotoxicity of CDDP, we explore the possible mechanisms involved. As CDDP generates ROS and *α*-M is an antioxidant compound, ROS production was measured. There was a tendency to increased ROS production (not reaching a statistical significance in relation to the control group) at a low concentration of CDDP or *α*-M alone, as well as with the combination treatment in the coincubation scheme (Figure 2). In the preincubation scheme, only the combination of 2 *μ*M CDDP plus 15 *μ*M *α*-M resulted in a significant increase of ROS generation with respect to the control group (Figure 3).

The fact that *α*-M herein stimulated CDDP-induced ROS production seems contrary to previous reports on this xanthone, which has been described as a scavenger of several ROS in a concentration-dependent manner [46]. Nevertheless, the 15 *μ*M concentration of *α*-M alone did not elevate ROS levels. According to Somasundaram and coworkers [47], the combination of nitric oxide donors and ROS generation may have a bifunctional response, prompting a pro- or antitumorigenic effect. The authors posed that this response depends on four elements: (1) the concentration of the NO donor and ROS inducers, (2) the treatment regimen, (3) the duration of treatment, and (4) the genetics of the cancer cells. Additionally, Halliwell [48] indicated that antioxidant agents might act as prooxidant compounds as part of a mixture (e.g., the mixture of *β*-carotene, ascorbate, and *α*-tocopherol). Hence, *α*-M may act as a prooxidant after previously functioning as an antineoplastic agent and then combined with CDDP at a low concentration.

The combination of *α*-M and CDDP decreased the viability of cervical cancer cells (Figure 1), which corresponds to an additive effect (Table 1). Apart from a rise in ROS levels (Figure 3), the combination of *α*-M and CDDP in the preincubation scheme caused a higher percentage of apoptosis. The present results are similar to those reported in the literature [13, 42] (Figure 5). Although in the coincubation scheme we did not find a significant difference between any of study groups, there is evidence in the literature indicating that *α*-M induces apoptosis in YD-15 tongue mucoepidermoid carcinoma cells starting at a concentration of 10 *μ*M [49].

In the preincubation scheme the combined treatment of *α*-M+CDDP herein prompted cell cycle arrest in the G2/M phase (Table 2), as did CDDP administered alone. Similar results were found in an oral squamous cell line after 24 h of exposure, without any changes detected at 48 h [41]. On the other hand, for *α*-M alone, we observed no changes in the cell cycle. However, it has been described that *α*-M significantly lengthens the duration of the G2/M phase in a colon cancer cell line [42], whereas in prostate cancer and pancreas cancer *α*-M induces arrest in G1 [13, 50]. So, the modification of the cell cycle by *α*-M depends on the cell line, concentration, and exposure time.

With the combination of the two compounds, *α*-M seems to decrease the cell cycle arrest of CDDP in the G2/M phase with the preincubation scheme, which may indicate interference by *α*M in the cytotoxic effect of CDDP. Nevertheless, the present data on cell viability and apoptosis show that the *α*M+CDDP combination kills the cancer cells. Hence, this combination probably activates other pathways, not necessarily involving alterations in the cell cycle. For example, it has been reported that *α*-M has effect on Ca^2+^-ATPase activity on endoplasmic reticulum and also causes a loss in the mitochondrial membrane potential and the release of cytochrome c, leading to apoptosis in PC12 cells [51]. Further experiments are needed to elucidate how a treatment based on *α*M and CDDP combination induces cell death.

After obtaining the current in vitro results, we proposed an experimental model of xenograft (Figure 6(a)). We demonstrated that the administration of *α*-M before CDDP delayed tumor growth, evidenced by a decrease in the final tumor volume. This antitumorigenic effect correlated with an increase in the cell doubling time in that group (Table 3), an outcome not accompanied by systemic toxicity (Figure 6(b)).

Systemic toxicity and renal toxicity do not necessarily parallel events. The kidney has the capacity to compensate for a loss of function when some of its nephrons are damaged. For this reason, in the present study we use some of early markers of kidney damage, such as BUN, urinary volume, urinary protein, and KIM-1. The current results confirm those found previously, in which *α*-M prove to protect the kidney from CDDP-induced damage (Figure 7) without modifying the pharmacokinetic of this drug [15]. It has previously been reported that *α*-M is effective against several types of cancer, including pancreas [50], prostate [13], and breast tumors [52]. We herein demonstrated for the first time that this compound is effective in the treatment of cervical cancer. Furthermore, evidence is presently provided in relation to the beneficial effect resulting from the combination of a natural product (*α*-M) and chemotherapy (CDDP) if the scheme of treatment is accurate.

In summary, the treatment combining *α*-M and CDDP led to distinct outcomes with a coincubation or preincubation scheme. The combined and simultaneous administration of *α*-M and CDDP caused a strong interaction between the two drugs and protection of the cancer cells. Contrarily, the administration of *α*-M before CDDP improved the therapeutic response exhibited by CDDP alone. This preincubation scheme limited tumor volume growth and augmented the cell doubling time without giving rise to secondary effects (systemic damage and/or nephrotoxicity). There was an increase in cell death, ROS production, and apoptosis, as well as the arrest of the cell cycle. Hence, *α*-M pretreatment increased CDDP toxicity without producing secondary effects. The current data suggest that *α*-M can be used as an adjuvant agent in those cancers whose treatment is based on CDDP. However, it is necessary to carry out clinical trials with patients to confirm the current findings.

## Figures and Tables

**Table 1:** Summary of the effect on HeLa cell of CDDP, *α*-M, and their combinations in the preincubation scheme. The table presents the combination index (CI) calculated based on the equation given in the text. ^*∗*^Mean values of three separate experiments performed in triplicate.

Drugs in combination		Drugs alone		Control growth (%)^*∗*^	Combination index	Interaction
CDDP *μ*M (*D*_1_)	*α*-M *μ*M (*D*_2_)	CDDP *μ*M (*D*_*x*1_)	*α*-M *μ*M (*D*_*x*2_)			
2	5	6.6	8.4	89.8	1.1	
2	10	20.7	13.8	73.0	0.8	Synergistic
5	10	25.4	14.8	68.0	1.0	Additive
2	15	37.9	16.5	56.1	1.0	Additive

**Table 2:** Cell cycle analysis after treatment with *α*-M, CDDP, and the combined treatments using both the coincubation and preincubation schemes. Values are the mean ± SD of three independent experiments for each point.

Groups	G1/G0	S	G2/M	G2/G1 ratio
Coincubation				
Control	59.1 ± 6.3	13.3 ± 4.3	27.6 ± 4.8	0.48 ± 0.12
CDDP 2 *μ*M	42.7 ± 8.3^a^	23.3 ± 7.4^a^	34.0 ± 5.8	0.83 ± 0.26
*α*-M 10 *μ*M	62.4 ± 7.1	12.3 ± 2.8	25.3 ± 4.8	0.42 ± 0.13
*α*-M 15 *μ*M	66.9 ± 6.3	10.6 ± 3.0	22.6 ± 5.8	0.35 ± 0.12
CDDP+*α*-M (2/10 *μ*M)	42.8 ± 2.1^a^	18.5 ± 4.2	38.7 ± 4.7^a^	0.91 ± 0.14
CDDP+*α*-M (2/15 *μ*M)	60.2 ± 8.7	13.8 ± 4.6	26.0 ± 5.9	0.45 ± 0.15

Preincubation				
Control	51.5 ± 1.7	11.9 ± 1.5	36.6 ± 2.2	0.71 ± 0.06
CDDP 2 *μ*M	29.8 ± 6.1^a^	20.6 ± 4.9^a^	53.8 ± 6.0^a^	1.86 ± 0.41
*α*-M 10 *μ*M	52.7 ± 7.3	12.0 ± 1.2	35.3 ± 3.8	0.70 ± 0.26
*α*-M 15 *μ*M	50.1 ± 4.7	11.3 ± 3.1	38.7 ± 2.4	0.78 ± 0.11
CDDP+*α*-M (2/10 *μ*M)	27.2 ± 5.8^a^	22.5 ± 6.5^a^	45.3 ± 7.2	1.73 ± 0.43
CDDP+*α*-M (2/15 *μ*M)	40.1 ± 11.2^a^	23.2 ± 5.1^a^	38.8 ± 8.7	1.17 ± 0.63

^a^ *p* ≤ 0.05  versus control group.

**Table 3:** Growth response of cervical cancer tumors treated with the combination of CDDP+*α*-M. Values are the mean ± SD of 6–8 mice per group.

	Tumor volume (mm^3^)		Cell doubling time (days)
	Initial	Final	
Control	168 ± 59	1132 ± 445	3.9 ± 1.2
CDDP	144 ± 41	712 ± 303^a^	4.7 ± 1.2^b^
*α*-M	143 ± 36	676 ± 325^a^	4.9 ± 2.3^c^
CDDP+*α*-M	192 ± 93	371 ± 233^a^	8.5 ± 4.3^a^

^a^versus control group; ^b^versus control CDDP; ^c^versus  *α*-M group; *p* ≤ 0.05.

## Additional Points

*Highlights*. Preincubation with *α*-M increased CDDP cytotoxicity in HeLa cultured cells. The combination of *α*-M+CDDP stemmed tumor growth. *α*-M avoids kidney damage without hindering with CDDP's antitumorigenic effect.
